# Investigation of the Stability of Human Freezing-Like Responses to Social Threat From Mid to Late Adolescence

**DOI:** 10.3389/fnbeh.2018.00097

**Published:** 2018-05-16

**Authors:** Hannah C. M. Niermann, Bernd Figner, Anna Tyborowska, Antonius H. N. Cillessen, Karin Roelofs

**Affiliations:** ^1^Behavioural Science Institute, Radboud University, Nijmegen, Netherlands; ^2^Donders Institute for Brain, Cognition and Behaviour, Radboud University, Nijmegen, Netherlands

**Keywords:** freezing-like behavior, stability, prospective longitudinal study, social threat, adolescence

## Abstract

Freezing behavior, a commonly observed defensive stress response, shows relatively high stability over time in animals. Given the relevance of freezing for stress-coping and human psychopathology, it is relevant to know whether freezing behavior is also stable in humans, particularly during adolescence, when most affective symptoms develop. In a prospective longitudinal study, we investigated freezing-like behavior in response to social threat in 75 adolescents at age 14, repeated 3 years later at age 17. We used a well-established method combining electrocardiography (ECG; heart rate) and posturography (body sway) in response to emotional picture-viewing of angry, happy, and neutral faces. We hypothesized that individual differences in freezing-like behavior in response to social threat—operationalized by contrasting angry vs. neutral faces—would be relatively stable over time. Our results indeed showed relative stability between ages 14 and 17 in individual differences in freezing-like behavior in heart rate (*r* = 0.82), as well as in combined heart rate and body sway measures (*r* = 0.65). These effects were not specific for the angry vs. neutral contrast; they were also visible in other emotion contrasts. Exploratory analysis in males and females separately showed stability in body sway specifically for angry vs. neutral faces only in females. Together, these results suggest moderate to strong stability in human freezing-like behavior in response to social threat from mid to late adolescence (with exception for the body sway measure in males). This relative stability was not specific for threat-induction and may reflect a general stability that is particularly strong for heart rate. The fact that this relative stability was found over a relatively long time range of 3 years is promising for studies aiming to use freezing-like behavior as a marker for internalizing symptoms in adolescent development.

## Introduction

Freezing is one of the main defensive responses to threat exposure, observed in various human and non-human species. It is characterized by bodily immobility and reduction in heart rate (Fanselow, 1984; Schenberg et al., 1993) and is generally thought to be an adaptive response by facilitating the selection of adequate coping responses, promoting perception, risk assessment and action preparation (Blanchard et al., 2011; Lojowska et al., 2015; Gladwin et al., 2016; Roelofs, 2017). However, deviations in freezing behavior—both in the form of absent or elevated levels of freezing—have been consistently associated with increased risk of developing internalizing symptoms (Bovin et al., 2008; Lopes et al., 2009; Adenauer et al., 2010; Roelofs et al., 2010; Kozlowska et al., 2015; Fragkaki et al., 2017; Niermann et al., 2017b; Niermann et al., submitted). Despite the increased interest in freezing behavior as a predictor for internalizing symptoms over the past few years, it remains unknown whether in humans, freezing behavior is a defensive response that is stable over time (Niermann et al., 2017a). In non-human species, threat-induced freezing has been observed to be relatively stable over time (Fox et al., 2008; Rogers et al., 2008). Testing whether freezing-like behavior is also stable in humans is particularly relevant for adolescence, a critical transition stage when individuals encounter many societal challenges, hormonal changes, and when most of the internalizing symptoms develop (Kessler et al., 2005; McLaughlin and King, 2015). Exposure to these challenges may increase adolescents’ risk to develop internalizing symptoms particularly in those adolescents who show altered freezing. The current study, therefore, tested the stability of freezing-like behavior in a prospective longitudinal investigation, following 75 adolescents from mid to late adolescence.

Upon threat exposure, both the sympathetic and parasympathetic branches of the autonomic nervous system (ANS) are activated, enabling fast onset of defensive freezing and fight-or-flight responses. Whereas freezing is associated with dominance in parasympathetic activity, fight-or-flight reactions are enabled by parasympathetic withdrawal and sympathetic elevation (Kozlowska et al., 2015; Roelofs, 2017). The freezing response in particular has been linked to optimal selection of and preparation for appropriate active responses. It has, for instance, been linked to enhanced visual processing of coarse visual features as well as to action preparation in active threat paradigms (Lojowska et al., 2015; Gladwin et al., 2016; Roelofs, 2017). Studies in several nonhuman species have demonstrated relative stability and heritability of freezing responses (Fox et al., 2008; Rogers et al., 2008), with freezing developing early in life and being stable and mature during adolescence (Moriceau et al., 2004). In line with this notion of freezing as a relatively stable response in animals, human freezing has been described as an important aspect of behavioral inhibition (Goldsmith et al., 1987; Buss et al., 2004). Behavioral inhibition—a trait characterized by shyness, withdrawal, and increased anxiety in novel situations—is often regarded to be relatively stable, particularly among individuals displaying extreme behaviors (Fox et al., 2005). However, whereas behavioral inhibition commonly entails a wide variety of behaviors, such as active withdrawal and expression of negative affect (Fox et al., 2005), the assessment of freezing as a separate type of behavior enables a specific exploration of a parasympathetically dominated state relevant for stress coping (Hermans et al., 2013; Hagenaars et al., 2014; Kozlowska et al., 2015; Lojowska et al., 2015; Roelofs, 2017).

So far, freezing-like behavior has been objectively quantified mainly in adults, using a well-established method combining electrocardiography (ECG) and posturography (e.g., Roelofs et al., 2010; Hagenaars et al., 2012). Only recently, it has been shown that these assessment methods can be used equally well to quantify freezing-like responses in developing populations, that are generally featured by lower body weight, which may affect the accuracy of subtle changes in body sway (Niermann et al., 2015, 2017b). Particularly adolescence—characterized by increased levels of internalizing symptoms (Kessler et al., 2005; McLaughlin and King, 2015)—is an important developmental period for investigating the stability of objectively quantified freezing responses in humans. Niermann et al. (2017b), for example, found that those adolescents who showed prolonged freezing responses 1 h after a standardized stress-induction, had increased levels of internalizing symptoms. Therefore, we set out to assess freezing-like behavior in a prospective longitudinal study at ages 14 and 17, using these well-established electrocardiographic and posturographic methods. The use of a stabilometric force-platform enabled us to assess subtle fluctuations in body sway combined with heart rate, in response to emotional picture-viewing of angry, happy and neutral faces. A long tradition of research has indicated that passive-viewing paradigms of aversive images—including angry faces as an indicator for social threat (Dimberg and Öhman, 1996; Öhman et al., 2001)—resemble an animal’s post-encounter stage of threat, with freezing as its main defensive stress response (Lang et al., 1997, 2000; Azevedo et al., 2005; Roelofs et al., 2010; Hermans et al., 2013; Niermann et al., 2015; Ly et al., 2017). This research line first used only electrocardiographic measures, but later combined them with posturographic measures. This research shows that reduction in heart rate in response to threat is significantly correlated to body sway reductions (Roelofs et al., 2010; Niermann et al., 2015, 2017b). In addition to this behavioral validation of freezing, neuroimaging studies demonstrated trial-by-trial correlations between threat-induced heart rate reductions and increased activity in neural defense structures, including the midbrain periaqueductal gray and its connection to the amygdala (Hermans et al., 2013). Based on these previous findings, we predicted that individual differences in freezing-like behavior in response to social threat, operationalized by contrasting responses to angry vs. neutral faces (Roelofs et al., 2010; Niermann et al., 2015) at age 14 would be positively associated with freezing-like behavior in response to the same emotion contrast assessed 3 years later at age 17.

## Materials and Methods

### Participants

Participants were recruited as part of the Nijmegen Longitudinal Study (NLS), which started with an original sample of 129 infants at 15 months of age and their families (van Bakel and Riksen-Walraven, 2002; Niermann et al., 2015, 2017b). Freezing data were assessed in 79 participants at age 14 (Niermann et al., 2015; *M*_age_ = 14.63, *SD* = 0.18, range: 14.33–15.17, 51% female) and in 96 participants at age 17 (Niermann et al., 2017b; *M*_age_ = 17.19, *SD* = 0.15, range: 16.83–17.70, 49% female). In total, 75 participants (49% female) yielded freezing data from both assessment points (due to technical problems, we do not have heart rate data for one of these participants at age 14, and for another participant we do not have body sway data at age 17). In accordance with the Declaration of Helsinki, participants and their parents provided written informed assent and written informed consent, respectively. They were paid for their participation. The study was approved by the local ethics committee (CMO region Arnhem-Nijmegen, Netherlands) and was carried out according to these guidelines. We preregistered the study at https://osf.io/y95qf/.

### Procedure and Measures

#### Emotional Face Task (EFT)

We assessed freezing behavior using an emotional face-viewing paradigm (Roelofs et al., 2010; Niermann et al., 2015, 2017b). To assess fluctuations in body sway, the Emotional Face Task (EFT) was administered while participants were standing on a stabilometric force platform. Simultaneously, ECG was recorded to assess heart rate: three heart rate electrodes were attached to the skin around the heart of the participants prior to testing. Next, we instructed participants to stand quietly on the stabilometric force platform and to passively watch happy, angry and neutral faces from 20 models (10 males and 10 females) of the Karolinska Directed Emotional Faces database (Lundqvist et al., 1988). Each model expressed each of the facial emotional expressions, which resulted in a total of 60 pictures (20 pictures per face category). The faces were presented in three blocks. Each block consisted of 20 face stimuli from the same emotional category (3 s presentation time for each face), which were presented consecutively without an inter-trial interval. Between blocks, there was an interval of 7 s (5 s black screen followed by 2 s white fixation cross). We randomized block and stimuli orders between participants. Each participant received the same block order at age 17 as he/she did at age 14. For more details regarding the instructions and the emotional face-viewing paradigm at age 14, see Niermann et al. (2015), and for more details at age 17, see Niermann et al. (2017b). For the current study, we only used the freezing assessment during the administration of the first EFT at age 17 as this was most comparable to the freezing assessment at age 14. The second and third EFTs at age 17 were preceded by a stress-induction procedure.

### Data Analysis

#### Posturography During EFTs

Four force sensors (one in each corner) of the stabilometric force platform recorded a time series of deviations from participants’ center-of-pressure (COP) in the anterior-posterior (AP) as well as the mediolateral (ML) directions (dimensions: 50 cm × 50 cm; sampling frequency: 200 Hz, 1 mm accuracy). We conducted posturographic analyses in MATLAB (MathWorks, Natick, MA, USA). We computed participants’ variability in body sway during each 1 min presentation block of facial emotional expressions from the same emotional category as an indicator for postural mobility, by determining the standard deviation of the COP in the AP direction (SD-AP; see Niermann et al., 2015 for a detailed description as well as Appendix S1 in Supplementary Online Material [SOM])^1^. Participants’ variability in body sway was adjusted for their weight.

#### Heart Rate During EFTs

For each of the facial emotional blocks of the EFT, we first manually determined participants’ heart rate peaks, using Brainvision (Analyzer 2.0). Participants’ heart rate in beats-per-minute (BPM) was then determined by counting the number of heart rate peaks for each of the 1-min facial emotional blocks of the EFT, using MATLAB (MathWorks, Natick, MA, USA)^1^.

#### Statistical Analyses^2^

All analyses were conducted in R (version 3.4.2; R Core Team, 2017). As an indicator for freezing behavior, we computed the difference score of heart rate in response to angry vs. neutral faces, with lower negative values indicating more freezing-like behavior in response to the angry (compared to neutral) faces. An identical difference score was calculated for body sway. This angry vs. neutral emotion contrast has been established previously as a meaningful contrast to illustrate individual differences in participants’ freezing behavior (Roelofs et al., 2010; Niermann et al., 2015, 2017b). The two indicators of freezing behavior (i.e., reductions in heart rate and body sway in response to angry vs. neutral faces) were tested separately.

To assess the relative stability in freezing behavior in response to angry vs. neutral faces, we computed correlations: (i) between the age 14 and age 17 heart rate difference scores of angry vs. neutral faces; and (ii) between the age 14 and age 17 analogous body sway difference scores. We computed Pearson correlations using the function *rcor.test* from the package *ltm* (Rizopoulos, 2006). As we tested the same hypothesis here with two correlational analyses (Rubin, 2017), we controlled for multiple testing using the false discovery rate via the *p.adjust* function from the *stats* package (R Core Team, 2017).

In the cases where we observed a significant correlation for our main emotion contrast of interest (i.e., angry vs. neutral), we ran equivalent follow-up analyses on the other emotion contrasts of heart rate and/or body sway (i.e., angry vs. happy, happy vs. neutral), as well as on heart rate and/or body sway in response to each emotion separately. This was done to determine the specificity of the observed results for our main emotion contrast of interest.

Because participants’ gender explained variations in individuals’ freezing-like behavior, we explored potential gender effects, by including gender not only as a main effect, but also as an interaction effect in regression analyses predicting freezing behavior to angry vs. neutral faces at age 17 from freezing behavior to the same emotion contrast at age 14 (*lavaan* package; using a robust estimator; Yves, 2012). Furthermore, we also explored the coherence between the two freezing measures (heart rate and body sway). We computed Pearson correlations between heart rate and body sway difference scores of angry vs. neutral faces separately at ages 14 and 17^3^, as well as between the averaged heart rate/body sway freezing-scores of angry vs. neutral faces across age. As for the analyses mentioned earlier, only in the cases where we observed significant results for these exploratory analyses for our main emotion contrast of interest, we ran equivalent follow-up analyses on the other emotion contrasts. Finally, we only report correlations corrected for multivariate outliers^4^ here, the uncorrected results are presented in SOM Appendices S4–S8. For our exploratory and follow-up analyses we did not control for multiple comparisons.

## Results

**Figure 1:**
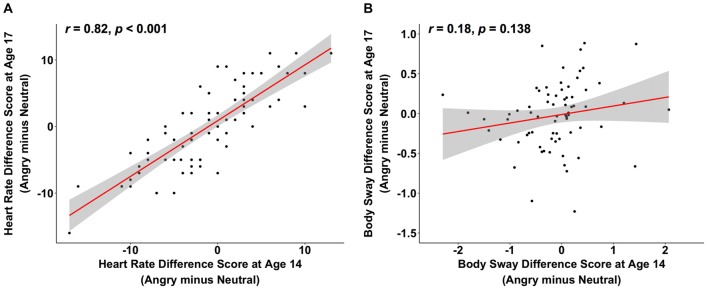
The scatterplots (with best-fitting regression line) illustrate the correlations between changes in heart rate variability (in beats per minute) at ages 14 and 17 **(A)** and between changes in body sway variability (in mm) at ages 14 and 17 **(B)** while participants were looking at angry compared to neutral faces. Three multivariate outliers were excluded for the correlations in **(A,B)**.

Reductions in heart rate in response to angry vs. neutral faces were positively correlated between ages 14 and 17, while the analogous body sway^5^ difference scores showed no such stability across time (heart rate: *r* = 0.82, *p* < 0.001, 95% CI [0.73, 0.89], body sway: *r* = 0.18, *p* = 0.138, 95% CI [−0.06, 0.39]; see Figure 1)^6^. Interestingly, when exploring the specificity of this positive association for the heart rate emotion contrast of angry vs. neutral faces, we observed similar significant correlations between ages 14 and 17 for the other emotion contrasts of heart rate (angry vs. happy: *r* = 0.77, *p* < 0.001, 95% CI [0.65, 0.85]; happy vs. neutral: *r* = 0.84, *p* < 0.001, 95% CI [0.76, 0.90]), as well as for heart rate in response to each emotion separately (angry: *r* = 0.57, *p* < 0.001, 95% CI [0.40, 0.71]; happy: *r* = 0.54, *p* < 0.001, 95% CI [0.35, 0.68]; neutral: *r* = 0.55, *p* < 0.001, 95% CI [0.36, 0.69]). Thus, freezing-like behavior as indicated by heart rate is relatively stable from mid to late adolescence and a similar pattern of stability was observed for other emotion contrasts. In addition, when comparing heart rate responses at ages 14 and 17, it is noteworthy that correlations in response to each emotion separately were numerically smaller than the correlations for the emotion contrasts.

### Exploratory Analyses

#### Gender

**Figure 2:**
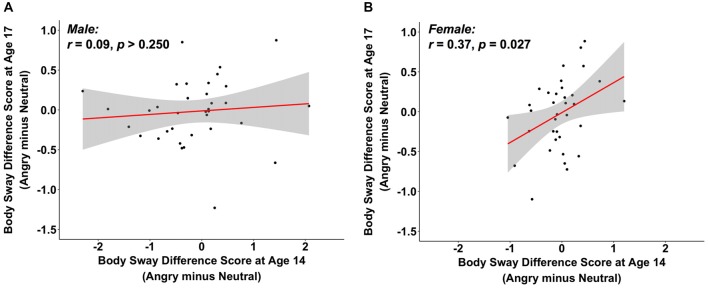
The scatterplots (with best-fitting regression line) illustrate the correlations between changes in body sway variability (in mm) at ages 14 and 17 to angry vs. neutral faces, separately for male **(A)** and female participants **(B)**. Two multivariate outliers were excluded for the correlations in **(A,B)**.

As there is evidence that anger might be differently processed by men and women (Kret and De Gelder, 2012), we explored potential gender effects by including gender as a main and as an interaction effect in a regression analysis, predicting heart rate difference scores for angry vs. neutral faces at age 17 from the same heart rate emotion contrasts at age 14. We observed a main effect of heart rate difference scores at age 14 (*B* = 0.84, *z* = 14.51, *p* < 0.001, 95% CI [0.73, 0.95]), but no main effect of gender (*B* = −1.38, *z* = −1.69, *p* = 0.091, 95% CI [−2.97, 0.22]) nor a moderation effect of gender (*B* = 0.02, *z* = 0.33, *p* > 0.250, 95% CI [−0.09, 0.13]; total *R*^2^ = 0.68). This supports the findings in the previous section that heart rate is generally stable, and equally so for both genders. Interestingly, when conducting the same regression analysis for the body sway difference score of angry vs. neutral faces, we observed no main effect of gender (*B* = 0.16, *z* = 1.34, *p* = 0.179, 95% CI [−0.08, 0.40]), but we did find a main effect of the body sway difference score of angry vs. neutral faces at age 14 (*B* = 0.19, *z* = 1.98, *p* = 0.048, 95% CI [0.001, 0.37]) as well as a moderation effect of gender (*B* = 0.22, *z* = 2.39, *p* = 0.017, 95% CI [0.04, 0.41]; total *R*^2^ = 0.09). To follow up on this moderation effect, we ran separate correlations for male and female participants for body sway difference scores of angry vs. neutral faces between ages 14 and 17. We observed a positive association for females (*r* = 0.37, *p* = 0.027, 95% CI [0.05, 0.63]), but not for males (*r* = 0.09, *p* > 0.250, 95% CI [−0.25, 0.41]; see Figures 2A,B). No significant associations were observed for the other emotion contrasts when correlations were computed separately by gender (Appendix S7 in SOM). This tentatively suggests that individual differences in body sway in reaction to angry vs. neutral faces may be stable for female but not for male participants.

#### Coherence Between Freezing Scores of Body Sway and Heart Rate

Because of the different stability patterns observed for heart rate and body sway, we explored whether these two freezing measures nevertheless showed a similar activation pattern, by associating them with each other at each age, as well as over time. Thus, we explored whether the participants who showed a reduction in heart rate in response to angry vs. neutral faces were also the participants who showed a reduction in body sway to angry vs. neutral faces. Indeed, the heart rate and body sway difference scores (of angry vs. neutral faces) were positively correlated with each other, both at age 14 as well as at age 17 (age 14: *r* = 0.30, *p* = 0.009, 95% CI [0.08, 0.49]; age 17: *r* = 0.35, *p* = 0.001, 95% CI [0.15, 0.51]). This is the same pattern as previously observed in Niermann et al. (2015, 2017b). Next, to investigate whether there was coherence between the two freezing measures (heart rate and body sway) over time, we computed an average freezing score per age. We did this by first standardizing heart rate and body sway difference scores of angry vs. neutral faces separately per age, and then averaging these scores per age. Due to technical problems, for two of our participants we had only body sway or only heart rate data. For these participants, we computed an adjusted average freezing-score by only using the non-missing observations. These average freezing-scores at ages 14 and 17 for angry vs. neutral faces were positively correlated (*r* = 0.65, *p* < 0.001, 95% CI [0.49, 0.76]).

Interestingly, similar associations were observed when correlating heart rate and body sway difference scores of *happy vs. neutral* faces separately at ages 14 and 17 (age 14: *r* = 0.24, *p* = 0.038, 95% CI [0.01, 0.44]; age 17: *r* = 0.21, *p* = 0.042, 95% CI [0.01, 0.40]), as well as when correlating the averaged heart rate/body sway freezing-scores of happy vs. neutral faces for each age (*r* = 0.59, *p* < 0.001, 95% CI [0.42, 0.72]). In contrast, heart rate and body sway difference scores of *angry vs. happy* faces were positively associated only at age 14 (*r* = 0.32, *p* = 0.005, 95% CI [0.10, 0.51]), but not at age 17 (*r* = 0.07, *p* > 0.250, 95% CI [−0.13, 0.27]). The averaged heart rate/body sway freezing-scores of angry vs. happy faces at ages 14 and 17 were positively correlated (*r* = 0.47, *p* < 0.001, 95% CI [0.27, 0.64]).

Taken together, these results suggest that there is coherence between the two freezing scores (heart rate and body sway) during mid as well as late adolescence and that there is stability over time in these combined freezing measures. Overall, these effects are not unique for the angry vs. neutral contrast; they are also present for the other emotion (happy vs. neutral) contrast.

## Discussion

The aim of this study was to investigate whether individual differences in human freezing-like behavior induced by social threat were relatively stable from mid to late adolescence. We objectively quantified freezing-like behavior as reductions in heart rate and body sway in response to angry vs. neutral faces. The results suggest that heart rate measures were generally stable between ages 14 and 17. Although heart rate and body sway in response to angry vs. neutral faces were positively correlated at each age as well as over time, stability of body sway to angry vs. neutral faces was only observed in females. Taken together, our findings suggest moderate to strong stability in human freezing-like behavior induced by social threat over a relatively long interval from mid to late adolescence (except for male body sway, which showed no stability). This relative stability was, however, not specific for threat-induction, and may reflect a general stability pattern for heart rate.

This is the first study investigating individual differences in objectively quantified freezing-like behavior in human adolescents and its stability over time. Approximately half of our participants showed a freezing-like pattern at each age, indicated by reductions in heart rate and/or body sway in response to angry vs. neutral faces (see Figures 1A,B). The remainder of the adolescents did not display a freezing-like pattern, but instead, showed no decrease, or even a relative increase in heart rate and/or body sway in response to angry vs. neutral faces. This is in line with previous research done in this adolescents sample, where at a group level, body sway reductions in response to angry vs. neutral faces were present at age 14 (Niermann et al., 2015), but not at age 17 (Niermann et al., 2017b), and where heart rate reductions were not present at the group level. The finding that not everyone shows freezing-like behavior in the current study is also in line with the great variability in animal’s freezing behavior, where some animals show fear bradycardia, while others show fear tachycardia (Hagenaars et al., 2014; Roelofs, 2017). In contrast to our current work, Niermann et al. (2015, 2017b), previous work in adults showed reduced heart rate as well as body sway in response to angry vs. neutral faces using the same EFT (Roelofs et al., 2010). These discrepancies may be related to several factors. First, adults have higher body weight and second, they show likely less movement in general. Both factors may affect the noise in the body sway measures. Besides these, there are several other factors that may have contributed to our findings. Adult studies, for instance, typically use stronger aversive stimuli to elicit robust and consistent freezing-like behavior, such as aversive pictures from the International Affective Picture System (IAPS) or threat of shock (Hagenaars et al., 2012; Hermans et al., 2013; Lojowska et al., 2015; Gladwin et al., 2016). Face stimuli, which we used in the current adolescent sample, may be weaker in eliciting robust freezing-like responses. Future research should determine whether stability of freezing-like behavior increases when freezing responses are induced in the context of stronger threat cues, such as IAPS pictures, or in the context of a fear-conditioning paradigm. The use of such stronger threat cues elicits freezing-like behavior in terms of heart rate and body sway reductions in about 80% of adult participants in our research group (Lojowska et al., 2015; Hashemi et al., 2016). Apart from these methodological considerations, however, we cannot rule out that freezing may show age differences such that individuals display less freezing at age 17 compared to age 14. Future research should establish whether a similar freezing stability pattern is observed when individuals are tested during a more stable phase of life that is not characterized by the profound developmental changes like the ones that take place between ages 14 and 17.

In line with previous research, heart rate was generally stable across time, irrespective of the emotion or emotion contrast (Cohen and Hamrick, 2003; Dragomir et al., 2014). While there was general stability in heart rate, the salient emotion contrast of main theoretical interest is the one of angry vs. neutral faces. Previous research (Roelofs et al., 2010; Niermann et al., 2015) suggests that heart rate differences between angry vs. neutral face stimuli are particularly indicative of freezing-like behavior because individual differences in this contrast were correlated with both individual differences in body sway in the analogous contrast and with internalizing symptoms. Therefore, it would be interesting for future research to examine the specificity of the emotion contrast of angry vs. neutral faces not only with respect to stability, but also with respect to the development of affective symptoms.

Interestingly for our body sway measure, exploratory analyses showed gender differences: Only female but not male participants showed stability in their body sway response to angry vs. neutral faces between ages 14 and 17. Compared to females, male participants showed more changes (i.e., less consistency) in their body sway response to angry vs. neutral faces from age 14 to age 17 (Figure 2). These findings may be explained in light of previously reported gender differences in the processing, experience, and expression of anger, with men scoring higher in identifying as well as expressing anger (for a review, see Kret and De Gelder, 2012). The gender effects found here were, however, part of exploratory analyses that were not adjusted for multiple comparisons and should be considered tentative. Future research is needed to more systematically investigate gender differences in freezing-like behavior in humans and its stability over time.

The current set-up did not allow us to disentangle an orienting response—involving an attentional component to orient towards a novel or threat stimuli—from a freezing response. Orienting is usually subjected to habituation (Hagenaars et al., 2014). However, our freezing measures showed no evidence for habituation when comparing the first 10 to the last 10 trials within a block (see Appendix S2 and Supplementary Table S1 in SOM), suggesting that we assessed a freezing instead of an orienting response in the current study. Nevertheless, it would be interesting for future research to increase block duration as this would allow for the assessment of heart rate variability, for instance by spectral analyses to detect activity in the parasympathetic nervous system. This might help to obtain a clearer characterization of the parasympathetic state associated with human freezing.

Although both body sway and heart rate are indices of freezing in humans, we observed interesting differences in the overall stability in heart rate and body sway, with larger overall stability over time in heart rate, compared to body sway. Differences in measurement error and measurement sensitivity of these posturographic and electrocardiographic methods may have contributed to the observed differences in stability. Furthermore, future longitudinal research should take the changes in adolescents’ height into account, because—like weight—height could influence the sensitivity of the body sway measure. Most critically, although both heart rate and body sway reductions during threat are generated by the midbrain periaqueductal gray, they follow different neural pathways, with the first projecting via the vagus nerve and the second via medullar projections to motor neurons in the spinal cord (Kozlowska et al., 2015; Roelofs, 2017). Thus, apart from methodological differences, these pathways have unique properties and may each develop within different time frames.

Despite these differences in stability in heart rate and body sway measures, we observed overall coherence between these two freezing measures at each age as well as over time. This was, however, not specific for angry vs. neutral faces; it was also observed for happy vs. neutral faces. It is tempting to speculate that this result is related to reports of adolescents’ (compared to adults’) increased responsiveness to incentives and emotional stimuli, such as happy faces (for a review see Somerville et al., 2010): for example, adolescents differ from adults in showing stronger ventral striatum and amygdala responses to happy relative to neutral facial expressions (Williams et al., 2006; Somerville et al., 2011). Similarly, early event-related potentials over the medial frontal regions in response to happy faces have been observed to weaken between adolescence and late adulthood (Williams et al., 2006). Based on these findings, we speculate that adolescents might show greater attention and emotional reactivity to happy facial expressions than adults, which may provoke a freezing-like pattern that is similar to responses to angry facial expressions. However, future research should systematically investigate whether a freezing-like response can also be observed in response to salience-inducing, positive stimuli, and whether it is meaningfully related to affective symptoms, similar to what has been observed for freezing-like responses to salience-inducing, negative stimuli (Roelofs et al., 2010; Niermann et al., 2015, 2017b).

Finally, one could argue that the observed coherence between the two freezing measures (heart rate and body sway) over time could be an artifact of the averaging procedure for calculating the freezing-score per age, combining reductions in heart rate and body sway. Therefore, the observed coherence may be driven by the general stability of heart rate in the current study. However, we found a significant positive association between reductions in heart rate and body sway at each age, which argues against this mathematical explanation fully accounting for the coherence of the two freezing measures of heart rate and body sway.

## Conclusion

In this prospective longitudinal study on the stability of freezing-like behavior, we observed moderate to strong stability in human freezing-like behavior in response to social threat from mid to late adolescence (except for male body sway, which did not show stability). However, this relative stability was not specific for social threat-induction and may reflect a general stability pattern, which is particularly pronounced for heart rate. The fact that this relative stability was found over a relatively long time range of 3 years is promising for studies aiming to use freezing-like behavior as a marker for internalizing symptoms in adolescent development.

## Data Availability

Data are available upon request from the Data Archiving and Networked Services (DANS): https://doi.org/10.17026/dans-z6v-r7tj

## Author Contributions

All authors contributed to the study concept and design. Data collection was done by HN and AT. HN performed data analysis and interpretation under the supervision of AC, BF and KR. HN drafted the article and all others provided critical revisions. All authors approved the final version of the article prior to submission.

## Conflict of Interest Statement

The authors declare that the research was conducted in the absence of any commercial or financial relationships that could be construed as a potential conflict of interest.

## Footnotes

^1^Exploratory analyses suggest no habituation over time within blocks, neither for body sway nor for heart rate difference scores (see Appendix S2 and Supplementary Table S1 in SOM).

^2^We also examined whether the previously observed association between insecure infant-parent attachment at 15 months of age and freezing behavior at age 14, established in this longitudinal data set (Niermann et al., 2015), extends to freezing behavior assessed in a similar way at age 17. In line with the proposed stability of freezing, we predicted that the experience of stress early in life (i.e., insecure infant-parent attachment and the experience of early negative personal life events) would have a similar positive association with freezing at ages 14 and 17, and that the experience of negative personal life events in-between freezing assessments (i.e., current stress) may reduce the proposed association of freezing behavior. However, we did not observe any influence of the experience of early and current stress on individual differences in freezing behavior and its stability over time. Additionally, no associations with state anxiety were observed. In the interest of shortness, the rationale, results, and conclusions of these analyses are reported in Appendix S3 and S9 in SOM (see also Supplementary Figures S1, S2 as well as Supplementary Tables S2, S3 in SOM).

^3^Data of all adolescents who participated in the assessments at either age 14 or age 17 were used to compute the correlations between heart rate and body sway difference scores separately per age.

^4^Multivariate outliers were determined based on a Mahalanobis distance >7.38 (Varmuza and Filzmoser, 2009), as previously used in Niermann et al. (2017b).

^5^As participants’ height may influence their body sway variability, we explored the influence of participants’ height on the assessment of body sway to angry vs. neutral faces (Berger et al., 1992; we could test this at age 17 only as we have no information of participants’ height at age 14). No association between height and body sway was observed (*r* = −0.05, *p* > 0.250, 95% CI [−0.26, 0.15]).

^6^Because of high variation in puberty at age 14, we explored whether participants’ puberty level at age 14 could explain variations in (stability of) freezing-like behavior, which was not the case (see Appendix S6 in SOM).
