# Supplementary material for: Investigation of the Stability of Human Freezing-Like Responses to Social Threat From Mid to Late Adolescence
Source: Front Behav Neurosci. 2018 May 16;12:97. doi: 10.3389/fnbeh.2018.00097 (PMC5964744; doi:10.3389/fnbeh.2018.00097)
Supplement: Supplementary file 1 [file Data_Sheet_1.docx]

***Supplemental Online Material***

**Investigation of the stability of human freezing-like responses to social threat from mid to late adolescence**

H.C.M. Niermann*, B. Figner, A. Tyborowska, A.H.N. Cillessen, & K. Roelofs

*** Correspondence:** Hannah C. M. Niermann: h.niermann@psych.ru.nl

**Appendix S1 – Posturography during Emotional Face Task**

Different from previously reported analyses, we used unfiltered and untransformed body sway data in this study. In Niermann et al. (2015) for the age 14 data, we used unfiltered, but log-transformed body sway data; in Niermann et al. (2017) for the age 17 data, we used filtered body sway data. Body sway data were handled slightly differently before at ages 14 and 17 to deal with noise in the data and to meet statistical assumptions. Importantly, we observed a similar result pattern for unfiltered and filtered (using a 10 Hz low-pass and a 0.1 Hz high-pass filter) body sway data. The exception is that gender effects were somewhat weaker for the filtered than for the unfiltered body sway data.

**Appendix S2 – Occurrence of habituation in heart rate and body sway?**

To explore whether habituation occurred across a block, we compared the heart rate difference scores computed for the presentation of the first 10 faces to the heart rate difference scores of the last 10 faces, separately for the 14-year-old data and the 17-year-old data. Towards this end, we ran two linear-mixed effect models (using the *lmer* function of the *lme4* package; Bates et al. (2015)), with heart rate difference scores as dependent variable at either age 14 or age 17. Each of these models had the following categorical predictors: (i) first 10 faces vs last 10 faces, (ii) emotion contrast (angry vs happy, angry vs neutral, happy vs neutral), and (iii) the interaction between (i) and (ii). *P*-values were determined using bootstrapped likelihood ratio tests (requested samples: 1000), using the function *mixed* of the package *afex* (Singmann et al., 2015). A significant interaction effect was of main interest for the occurrence of habituation. However, we observed no interaction effect for age 14 or age 17, indicating no habituation of heart rate in the last compared to the first trials within a block (see Table S1 in SOM). We ran the same linear-mixed effect models, separately for age 14 and age 17, but this time with body sway difference scores as dependent variable. Again, we observed no interaction effect, indicating also for body sway no habituation in the last compared to the first 10 trials within a block (see Table S1). These results suggest that indeed there was freezing over the whole block, without evidence for habituation within a block.

Table S1. *The Effects of First 10 vs Last 10 Trials, Emotion Contrast, and their Interactions on Body Sway and Heart Rate Difference Scores.*

|  | Model: Heart rate difference scores | | Model: Body sway difference scores | |
| --- | --- | --- | --- | --- |
| Effect | χ^2^ (df) | *p* | χ^2^ (df) | *p* |
| **Age 14** |  |  |  |  |
| First 10 vs last 10 trials | χ^2^ (1) = 4.28 | .037 | χ^2^ (1) = 8.61 | .004 |
| Emotion contrast | χ^2^ (2) = 5.30 | .055 | χ^2^ (2) = 4.42 | .097 |
| First 10 vs last 10 trials × Emotion contrast | χ^2^ (2) = 0.75 | >.250 | χ^2^ (2) = 1.45 | >.250 |
|  |  |  |  |  |
| **Age 17** |  |  |  |  |
| First 10 vs last 10 trials | χ^2^ (1) = 0.07 | >.250 | χ^2^ (1) = 0.05 | >.250 |
| Emotion contrast | χ^2^ (2) = 1.52 | >.250 | χ^2^ (2) = 0.33 | >.250 |
| First 10 vs last 10 trials × Emotion contrast | χ^2^ (2) = 0.64 | >.250 | χ^2^ (2) = 0.08 | >.250 |

**Appendix S3 – Early and Current Stress**

**Rationale.** Despite the proposed relative stability of individual differences in animals’ freezing responses, some flexibility in freezing behavior is necessary to adapt successfully to environmental changes. Indeed, contextual changes (Imanaka et al., 2006), exposure to severe events (van Dijken et al., 1992; van den Berg et al., 1998; Daviu et al., 2010), and chronic stress (Conrad et al., 1999) have been shown to alter animals’ freezing response. Additionally, research in primates and rodents has demonstrated that the experience of early life stress—such as low levels of maternal licking and grooming or postnatal separation from the mother—can result in long-lasting and enduring changes in animals’ stress coping behavior across the lifetime. This includes increased levels of freezing behavior, life-long anxious, depressive, and avoidant behavior, and maladaptive stress coping (Menard et al., 2004; Imanaka et al., 2006; Sanders and Knoepfler, 2008; Lukkes et al., 2009; Rincón-Cortés and Sullivan, 2016; Liu et al., 2017; Yan et al., 2017). In an effort to extend this animal work to humans, human freezing behavior has been shown to be sensitive to the experience of life events, state anxiety, acute stress, as well as a history of insecure infant-parent attachment (Roelofs et al., 2010; Hagenaars et al., 2012; Niermann et al., 2015; Niermann et al., 2017).

**Hypotheses.** We hypothesized that the experience of negative personal life events in between freezing assessments (i.e., current stress) may reduce the proposed association of freezing behavior. We also investigated whether the previously observed positive association between insecure infant-parent attachment at 15 months of age and freezing behavior at age 14, established in this longitudinal data set (Niermann et al., 2015), extends to freezing behavior assessed in a similar way three years later at age 17. In line with the proposed stability of freezing behavior, we predicted that if freezing is relatively stable, the experience of early stress should have a similar positive association with freezing at ages 14 and 17. We also hypothesized that—in addition to insecure infant-parent attachment—early negative personal life events will have similar effects on freezing behavior at ages 14 and 17.

**Assessment of Infant-Parent Attachment (15 months of Age).** Quality of infant-parent attachment was assessed in the 15-month wave of the Nijmegen Longitudinal Study, using an abbreviated version of the Strange Situation Procedure (Ainsworth et al., 1978). This assessment procedure is described in detail by van Bakel and Riksen-Walraven (2002). To classify infants as Secure (B), Avoidant (A), Resistant (C), or Disorganized and Disoriented (D), we used directions provided by Ainsworth et al. (1978) and Main and Solomon (1990). The inter-rater reliability determined from a subset of 20 randomly selected infants was 95% (van Bakel and Riksen-Walraven, 2002). For the current study, we only categorized individuals as securely (i.e., category B; *n*=68) or insecurely (i.e., categories A, C, D; *n*=32) attached.

**Assessment of Early and Current Negative Personal Life Events.** We assessed the experience of early negative personal life events (before age 5) and current negative personal life events (between ages 14 and 17) via parent reports within one to two years after the event had taken place. Parents were asked to indicate with *yes* or *no* whether their child had experienced one or more stressful life events. Items from Saranson et al. (1978) Life Experiences Survey and from Coddington (1972) Life Events Scale for Children were selected that were likely to have a negative influence on participants’ development (e.g., divorce, death of a loved one, serious illness of child or parent). The final scores represent the total number of negative life events the participants had experienced in early childhood (before age 5) and during adolescence (between ages 14 and 17).

**Statistical analyses.** The indicator for freezing behavior was determined in the same way as described in the main text, namely as a difference score of heart rate in response to angry vs neutral faces, with lower negative values indicating more freezing-like behavior. An identical difference score was calculated for body sway. Both indicators of freezing-like behavior (i.e., reduction in heart rate and body sway in response to angry vs neutral faces) were tested separately. To test the influence of early and current stress on freezing behavior, we used the *lavaan* package in R (Yves, 2012), with full information maximum likelihood estimation to deal with missing values^[[1]](#footnote-1)^. To investigate whether the experience of current stress (i.e., personal negative life events) moderates the proposed stability between freezing in response to angry vs neutral faces during adolescence, we conducted regression analyses using a robust estimator (Figure S1). We ran two regression models, one with reduction in heart rate, and one with reduction in body sway in response to angry vs neutral faces at age 17 as the dependent variable. Both models had the same predictors: (i) the reduction in heart rate (for the heart rate model) or body sway (for the body sway model) in response to angry vs neutral faces at age 14, (ii) negative personal life events (age 14-17), and (iii) the interaction between (i) and (ii).


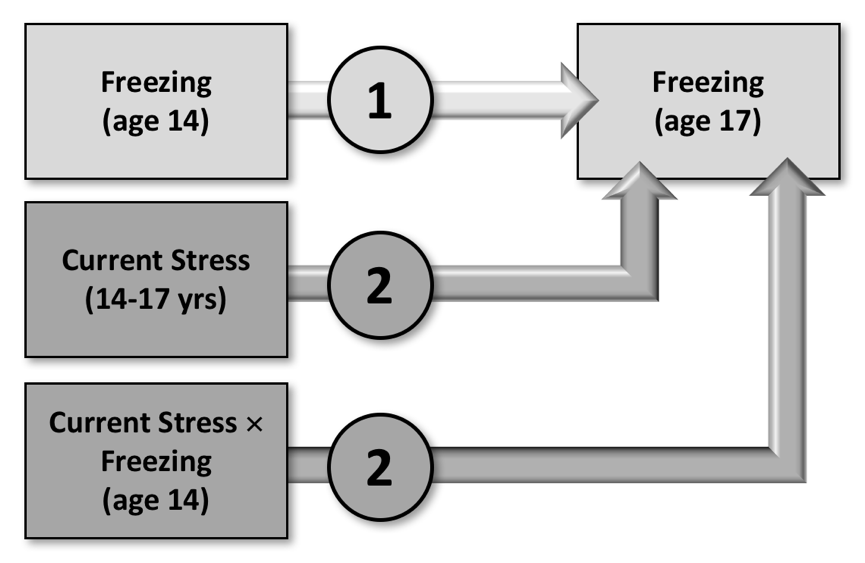


**Figure S1.** Regression model to test (1) the relative stability of freezing-like behavior in response to angry vs neutral faces from age 14 to age 17, and (2) the main and moderating effect of current stress (age 14-17)—characterized by negative personal life events—on the association of freezing-like behavior in response to angry vs neutral faces between ages 14 and 17.

To test whether early life stress (i.e., insecure infant-parent attachment and/or negative personal life events) predicted freezing-like behavior in response to angry vs neutral faces assessed at ages 14 and 17, we ran structural equation models using bootstrapping (see Figure S2). We investigated the effect of infant-parent attachment security by running two structural equation models, one with reduction in heart rate and one with reduction in body sway in response to angry vs neutral faces at age 17 as the dependent variable. Otherwise, both models included (i) infant-parent attachment security as a predictor, and (ii) reduction in heart rate (for the heart rate model) or body sway (for the body sway model) in response to angry vs neutral faces at age 14 as a mediator. To test the effect of early negative personal life events, we ran two additional structural equation models that were identical to the ones just reported, except for the inclusion of early negative personal life events instead of infant-parent attachment security as a predictor. In these four models, we tested the predictive value of early life stress (i.e., insecure infant-parent attachment or negative personal life events) on freezing-like behavior at age 14. Additionally, in these models we determined the extended effect of early life stress on freezing-like behavior at age 17 by adding a direct and indirect path via freezing behavior at age 14, testing for mediation.


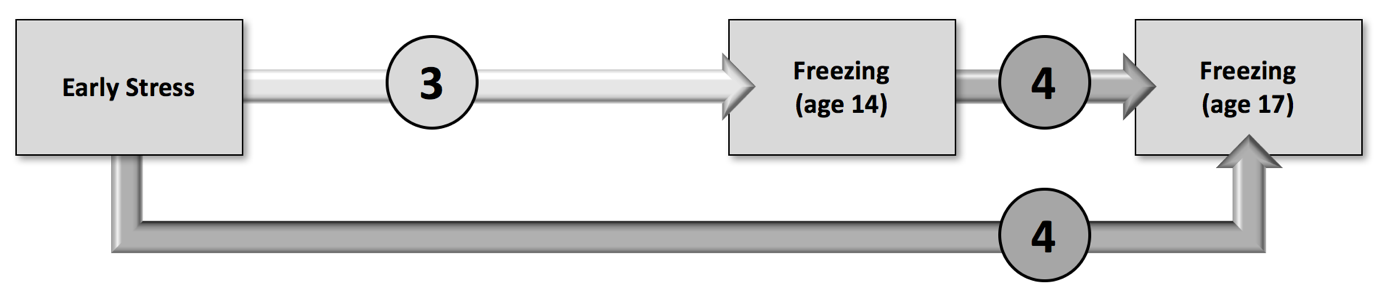


**Figure S2.** Structural equation model to test (3) the predictive value of early life stress—characterized in separate models for insecure infant-parent attachment and for negative personal life events (0-5 years)—on freezing-like behavior in response to angry vs neutral faces at age 14, and to determine (4) the extended effect of early life stress on freezing-like behavior in response to angry vs neutral faces at age 17 by adding a direct and indirect path via freezing behavior at age 14, testing for mediation.

As we tested the same hypothesis with more than one analysis (Rubin, 2017), we controlled for multiple testing using the false discovery rate via the *p.adjust* function from the *stats* package (R Core Team, 2017). To better understand the results, we explored gender effects, by adding gender as a main effect to the models. Additionally, in the cases where we observed significant coefficients for our main emotion contrast of interest (i.e., angry vs neutral), we ran equivalent follow-up analyses on the other emotion contrasts of heart rate and/or body sway (i.e., angry vs happy, happy vs neutral), as well as on heart rate and/or body sway in response to each emotion separately^[[2]](#footnote-2)^.

**Results for Current Stress on Freezing.** We observed no moderation effect for the experience of current stress—in the form of negative personal life events—on the stability of freezing-like behavior, expressed as reduction in heart rate and body sway in response to angry vs neutral faces at ages 14 and 17 (see Table S2). These results remained when controlling for gender. When conducting follow-up regression models for the other emotion contrasts of heart rate as well as for heart rate in response to each emotion separately, we also observed no moderation effects for current stress. Similarly as reported for the correlational analyses in the main text, we observed in each of these models a positive predictive value of heart rate at age 14 on heart rate at age 17 (irrespective of emotion and emotion contrast; all *p*s < .001).

Table S2.

*Results of the Multiple Regression Analyses Investigating the Moderating Effect of Current Stress on the Stability of Freezing-like Behavior at Ages 14 and 17.*

|  | ***B*** | ***SE B*** | ***β*** | ***z*** | ***p_uncor_*** | ***p_cor_*** | ***R^2^*** |
| --- | --- | --- | --- | --- | --- | --- | --- |
| **HR reduction (Age 17)** |  |  |  |  |  |  | 0.66 |
| HR reduction (Age 14) | 0.81 | 0.06 | 0.80 | 14.34 | <.001 | <.001 |  |
| Current Stress | 0.03 | 0.27 | 0.01 | 0.12 | >.250 | >.250 |  |
| HR reduction × Current Stress | -0.06 | 0.05 | -0.07 | -1.17 | .243 | >.250 |  |
|  |  |  |  |  |  |  |  |
| **BS reduction (Age 17)** |  |  |  |  |  |  | 0.01 |
| BS reduction (Age 14) | 0.05 | 0.07 | 0.07 | 0.61 | >.250 | >.250 |  |
| Current Stress | -0.04 | 0.04 | -0.10 | -0.96 | >.250 | >.250 |  |
| BS reduction × Current Stress | 0.00 | 0.05 | 0.00 | 0.00 | >.250 | >.250 |  |

*Note.* *p_uncor_* = uncorrected *p*-values; *p_cor_* = corrected *p*-values. Freezing behavior is expressed as reductions in heart rate (HR) and body sway (BS) in response to angry vs neutral faces.

**Results for Early Life Stress on Freezing.** We found neither a direct nor an indirect effect of early life stress on freezing-like behavior in response to angry vs neutral faces at ages 14 and 17 (see Table S3). Again, these results did not change when controlling for gender. When conducting follow-up structural equation models for the other emotion contrasts of heart rate as well as for heart rate in response to each emotion separately, we also observed no effect of early life stress. Similar as reported above, we observed in each of these models a positive predictive value of heart rate at age 14 on heart rate at age 17 (irrespective of emotion and emotion contrast; all *p*s < .001).

Table S3.

*Results of the Structural Equation Models Investigating the Direct and Indirect Effect of Early Stress on Freezing Behavior at Ages 14 and 17.*

|  | ***B*** | ***SE B*** | ***β*** | ***z*** | ***p_uncor_*** | ***p_cor_*** | ***R^2^*** |
| --- | --- | --- | --- | --- | --- | --- | --- |
| **HR reduction (Age 17)** |  |  |  |  |  |  | 0.66 |
| Attachment | 0.43 | 0.84 | 0.03 | 0.52 | >.250 | >.250 |  |
| HR reduction (Age14) | 0.82 | 0.06 | 0.81 | 13.41 | <.001 | <.001 |  |
| Indirect | -0.86 | 1.13 | -0.07 | -0.75 | >.250 | >.250 |  |
| **HR reduction (Age 14)** |  |  |  |  |  |  | 0.01 |
| Attachment | -1.04 | 1.36 | -0.08 | -0.76 | >.250 | >.250 |  |
|  |  |  |  |  |  |  |  |
| **HR reduction (Age 17)** |  |  |  |  |  |  | 0.66 |
| Early Stress | -0.32 | 0.33 | -0.06 | -0.98 | >.250 | >.250 |  |
| HR reduction (Age14) | 0.82 | 0.06 | 0.81 | 12.96 | <.001 | <.001 |  |
| Indirect | -0.27 | 0.50 | -0.05 | -0.53 | >.250 | >.250 |  |
| **HR reduction (Age 14)** |  |  |  |  |  |  | 0.00 |
| Early Stress | -0.33 | 0.61 | -0.06 | -0.54 | >.250 | >.250 |  |
|  |  |  |  |  |  |  |  |
| **BS reduction (Age 17)** |  |  |  |  |  |  | 0.02 |
| Attachment | -0.16 | 0.11 | -0.15 | -1.45 | .148 | >.250 |  |
| BS reduction (Age14) | 0.05 | 0.09 | 0.07 | 0.52 | >.250 | >.250 |  |
| Indirect | 0.02 | 0.04 | 0.02 | 0.58 | >.250 | >.250 |  |
| **BS reduction (Age 14)** |  |  |  |  |  |  | 0.09 |
| Attachment | 0.53 | 0.24 | 0.30 | 2.23 | .025 | .100 |  |
|  |  |  |  |  |  |  |  |
| **BS reduction (Age 17)** |  |  |  |  |  |  | 0.01 |
| Early Stress | 0.04 | 0.05 | 0.08 | 0.72 | >.250 | >.250 |  |
| BS reduction (Age14) | 0.02 | 0.08 | 0.04 | 0.30 | >.250 | >.250 |  |
| Indirect | 0.00 | 0.01 | 0.00 | 0.19 | >.250 | >.250 |  |
| **BS reduction (Age 14)** |  |  |  |  |  |  | 0.00 |
| Early Stress | 0.05 | 0.06 | 0.07 | 0.78 | >.250 | >.250 |  |

*Note.* *p_uncor_* = uncorrected *p*-values; *p_cor_* = corrected *p*-values. Freezing behavior is expressed as reductions in heart rate (HR) and body sway (BS) in response to angry vs neutral faces.

**Conclusions.** Contrary to research in animals, we found no influence of early or current stress on individual differences in freezing behavior and its stability over time. In the current study, early and current negative personal events may not have been severe enough to elicit changes in an individuals’ freezing response. Future research should systematically investigate the influence of various life events, varying in their level of adversity, on individuals’ freezing behavior and its stability. Importantly, we could not replicate the previously observed association between insecure infant-parent attachment and increased adolescents’ bodily freezing behavior at age 14 (Niermann et al., 2015), again at age 17. There are several potential explanations for this unexpected finding. First, insecure infant-parent attachment may no longer have an effect on freezing-like behavior during late adolescence. Second, the previously observed association between infant-parent attachment security and later bodily freezing-like behavior at age 14 might have been a false positive. However, we regard this as unlikely as research in animals clearly supports this longitudinal association (Menard et al., 2004; Imanaka et al., 2006; Sanders and Knoepfler, 2008; Lukkes et al., 2009; Rincón-Cortés and Sullivan, 2016; Liu et al., 2017; Yan et al., 2017). Nevertheless, future longitudinal investigation should replicate this association in an independent sample. Third, the absence of an association between insecure infant-parent attachment and adolescents’ freezing-like behavior at age 17 may also have been due to the stability pattern of freezing-like behavior observed in this study. Previously, we found only a main effect of reductions in body sway, but not in heart rate, to angry vs neutral faces at age 14 (Niermann et al., 2015), but none at age 17 (Niermann et al., 2017). This may suggest that the freezing measurement at age 17 was somewhat noisier than the measurement at age 14, which can potentially be explained by differences in measurement context. The anticipation of the stress-induction procedure used at age 17 may have obscured the salience of a picture depicting an angry face. Therefore, it is possible that the current study was not able to reliably assess individual differences in freezing-like behavior to social threat over time (see also the discussion section in the main text), potentially explaining why we were not able to establish an association between insecure infant-parent attachment and bodily freezing again at age 17.

**Appendix S4 –Main Correlational Results for Heart Rate and Body Sway, Based on All Participants**

Similar results as reported in the main text were observed when correlational results were not corrected for multivariate outliers (i.e., correlations were computed based on all participants): only reductions in heart rate, but not in body sway, in response to angry vs neutral faces were positively correlated between ages 14 and 17 (heart rate: *r* = .82, *p* < .001, 95% CI [.73, .88]; body sway: *r* = .05, *p* > .250, 95% CI [-.18, .27]; 3 and 3 multivariate outliers were excluded respectively for the corresponding correlations reported in the main text). Again similar as reported in the main text, correlational results, including all participants, revealed similar significant associations between ages 14 and 17 for the other emotion contrasts of heart rate (angry vs happy: *r* = .77, *p* < .001, 95% CI [.65, .85]; happy vs neutral: *r* = .79, *p* < .001, 95% CI [.69, .86]; 3 and 4 multivariate outliers were excluded respectively for the corresponding correlations reported in the main text), as well as for heart rate in response to each emotion separately (angry: *r* = .53, *p* < .001, 95% CI [.35, .68]; happy: *r* = .54, *p* < .001, 95% CI [.35, .68]; neutral: *r* = .55, *p* < .001, 95% CI [.36, .69]; 1, 2, and 0 multivariate outliers were excluded respectively for the corresponding correlations reported in the main text).

**Appendix S5 – Results of Exploratory Analyses of Correlation between Body Sway Difference Score of Angry vs Neutral Faces and Height, Based on All Participants**

Similar non-significant association between body sway difference scores of angry vs neutral faces and participants’ height at age 17—as reported in the main text—were observed when multivariate outliers were not excluded (*r* = .02, *p* > .250, 95% CI [-.18, .22]; 3 multivariate outliers were excluded for the corresponding correlation reported in the main text). Data of all 96 adolescents who participated at the age 17 assessment wave were used to compute this correlation.

**Appendix S6 – Puberty at age 14**

Because of high variation in puberty at age 14, we explored whether participants’ level of puberty at age 14 could explain variations in (stability of) freezing-like behavior. As an indicator for the participants’ level of puberty at age 14, we used the average score of the Pubertal Development Scale, separately calculated for males and females (PDS; Petersen et al., 1988), as well as their testosterone levels, assessed via saliva prior to the freezing assessment. As was previously done within our research group, testosterone values were first log-transformed and then standardized within gender (Tyborowska et al., 2016). We correlated these two indicators of puberty at age 14 with the freezing assessment at the same age, separately indicated by body sway and heart rate reduction in response to angry vs neutral faces. We found no association between participants’ level of puberty and their level of freezing-like behavior, expressed as reductions in heart rate (PDS: *r* = -.20, *p* = .092, 95% CI [-.41, .03], testosterone: *r* = .05, *p* > .250, 95% CI [-.23, .32]) and body sway (PDS: *r* = .07, *p* > .250, 95% CI [-.16, .29], testosterone: *r* = -.11, *p* > .250, 95% CI [-.37, .16]). When excluding multivariate outliers in these correlational analyses (i.e., Mahalanobis distance > 7.38; Varmuza and Filzmoser (2009)), the results remained the same except that the negative association between level of puberty, indicated by PDS, and reductions in heart rate in response to angry vs neutral faces became significant (exclusion of 4 multivariate outliers: *r* = -.30, *p* = .011, 95% CI [-.50, -.07]). Based on this result, we explored whether level of puberty at age 14, indicated by PDS, could explain the association between reductions in heart rate in response to angry vs neutral faces between ages 14 and 17. Similar to previous analyses, we used the *lavaan* package in R (Yves, 2012)—with full information maximum likelihood estimation to deal with missing values—to conduct a regression analysis using a robust estimator. The regression model included reduction in heart rate in response to angry vs neutral faces at age 17 as the dependent variable, and the reduction in heart rate in response to angry vs neutral faces at age 14, and participants’ level of puberty at age 14 (indicated by PDS) as predictors. The results suggested that level of puberty could not explain the association between reductions in heart rate in response to angry vs neutral faces across age (R^2^ = .66, heart rate reduction at age 14: *B* = 0.82, *z* = 13.55, *p* < .001, 95% CI [0.70, 0.94]; PDS: *B* = 0.00, *z* = -0.01, *p* > .250, 95% CI [-1.11, 1.10]).

**Appendix S7 – Gender Differences for Body Sway Difference Scores**

In the main text, we reported a regression model that tested the moderation effect of gender on the associations between body sway difference scores of angry vs neutral faces at ages 14 and 17. To follow-up on the observed moderation effect, we reported in the main text separate correlations for male and female participants for this body sway emotion contrast, which were corrected for multivariate outliers. When we did not correct for multivariate outliers, similar correlations were found. Specifically, we observed a positive association for female participants (*r* = .40, *p* = .014, 95% CI [.09, .64]; 2 multivariate outliers were excluded for the reported correlation in the main text), but not for male participants (*r* = -.08, *p* > .250, 95% CI [-.39, .25]; 2 multivariate outliers were excluded for the reported correlation in the main text). Similar as reported in the main text, this tentatively suggests that individual differences in body sway to angry vs neutral faces may be stable for female but not for male participants.

To determine the specificity of this moderation effect of gender for the body sway difference scores of angry vs neutral faces between ages 14 and 17, as reported in the main text, we conducted the regression analysis again, but this time in response to the other emotion contrasts. Gender moderated the association between body sway difference scores of angry vs happy faces between ages 14 and 17 (*R^2^* = 0.12; body sway to angry vs happy faces [age 14]: *B* = -0.11, *z* = -1.17, *p* = .240, 95% CI [-0.29, 0.07]; gender: *B* = 0.04, *z* = 0.46, *p* > .250, 95% CI [-0.13, 0.21]; gender × body sway to angry vs happy faces [age 14]: *B* = -0.27, *z* = -2.90, *p* = .004, 95% CI [-0.45, -0.09]), but not between body sway difference scores of happy vs neutral faces between ages 14 and 17 (*R^2^* = 0.04; body sway to happy vs neutral faces [age 14]: *B* = 0.09, *z* = 0.94, *p* > .250, 95% CI [-0.09, 0.26]; gender: *B* = 0.09, *z* = 0.76, *p* > .250, 95% CI [-0.14, 0.31]; gender × body sway to happy vs neutral faces [age 14]: *B* = 0.15, *z* = 1.69, *p* = .091, 95% CI [-0.02, 0.33]). To follow up on this moderation effect of gender on the body sway difference scores of angry vs happy faces between ages 14 and 17, we ran separate correlations for male and female participants. After multivariate outlier correction, we observed neither for male nor for female participants a significant association for the body sway difference scores of angry vs happy faces between ages 14 and 17 (male: *r* = .34, *p* = .039, 95% CI [.02, .60]; exclusion of 3 multivariate outliers: *r* = .26, *p* = .140, 95% CI [-.09, .55]; female: *r* = -.35, *p* = .034, 95% CI [-.60, -.03]; exclusion of 1 multivariate outlier: *r* = -.12, *p* > .250, 95% CI [-.43, .21]). This suggests that male and female participants significantly differed from each other when associating body sway difference scores of angry vs happy faces between ages 14 and 17. However, when determining these correlations of body sway difference scores to angry vs happy faces between ages 14 and 17 separately for male and female participants—as a follow-up analysis—they did not differ from zero.

**Appendix S8 – Coherence Between Two Freezing Measures, Based on All Participants^[[3]](#footnote-3)^**

Similar results were observed when multivariate outliers were not excluded for correlational analyses exploring the coherence between the two freezing measures of body sway and heart rate. Indeed, heart rate and body sway difference scores of angry vs neutral faces were (marginal significantly) positively correlated at ages 14 and 17 (age 14: *r* = .19, *p* = .100, 95% CI [-.04, .39]; age 17: *r* = .29, *p* = .004, 95% CI [.09, .46]; 1 and 3 multivariate outliers were excluded respectively in corresponding correlations reported in the main text). Similarly, the average freezing-scores of combined heart rate and body sway difference scores to angry vs neutral faces at ages 14 and 17 were positively correlated (*r* = .55, *p* < .001, 95% CI [.37, .69]; 2 multivariate outliers were excluded in corresponding correlation reported in the main text).

Similar as reported in the main text, when including all participants we also observed (marginal significantly) positive associations between heart rate and body sway differences scores of happy vs neutral faces at ages 14 and 17 (age 14: *r* = .20, *p* = .082, 95% CI [-.03, .40]; age 17: *r* = .29 *p* = .004, 95% CI [.09, .46]; 3 and 3 multivariate outliers were excluded respectively in corresponding correlations reported in the main text) as well as when correlating the averaged heart rate/body sway freezing-scores of happy vs neutral faces across age (*r* = .51, *p* < .001, 95% CI [.31, .66]; 2 multivariate outliers were excluded in the corresponding correlations in the main text).

Again, similar as in the main text, when including all participants, heart rate and body sway difference scores of angry vs happy faces were positively associated only at age 14 (*r* = .38, *p* = .001, 95% CI [.18, .56]; 4 multivariate outliers were excluded in the corresponding correlation in the main text), but not at age 17 (*r* = .06, *p* > .250, 95% CI [-.14, .26]; 1 multivariate outlier was excluded in the corresponding correlation reported in the main text). The averaged heart rate/body sway freezing-scores of angry vs happy faces at ages 14 and 17 were positively correlated (*r* = .50, *p* < .001, 95% CI [.30, .65]; 4 multivariate outliers were excluded in the corresponding correlation in the main text).

**Appendix S9 – State Anxiety**

State anxiety was assessed before the freezing assessment at both ages 14 and 17. We used a self-report visual analog (VAS) scale on which participants indicated on a scale from 0–100 (0 = *not anxious at all*; 100 = *extremely anxious*) how anxious they felt at that moment. Data at age 14 were missing for four participants; for three of them, we relied on a previous assessment filled in approximately 1h before.

To help interpret the observed coherence in heart rate and body sway to angry vs neutral faces as well as to happy vs neutral faces, in terms of whether they reflect freezing-like behavior, we explored whether heart rate and/or body sway difference scores of these two emotion contrasts were associated with individuals’ level of self-reported state anxiety, separately at ages 14 and 17. As previous research suggests that state anxiety is only associated with freezing-like behavior in response to angry faces (Roelofs et al., 2010; Niermann et al., 2015), we expected that we would observe a negative association for angry vs neutral faces, but not for happy vs neutral faces. However, we observed no associations with state anxiety, neither with the difference scores of angry vs neutral faces nor with the differences score of happy vs neutral faces (all *p*s > .05).

**References**

Ainsworth, M.D.S., Blehar, M.C., Waters, E., and Wall, S. (1978). *Patterns of attachment: A psychological study of the strange situation.* Hillsdale, NJ: Erlbaum.

Bates, D., Maechler, M., Bolker, B., and Walker, S. (2015). Fitting linear mixed-effects models using lme4. *Journal of Statistical Software* 67(1)**,** 1-48. doi: 10.18637/jss.v067.i01.

Coddington, R. (1972). The significance of life events as etiological factors in the diseases of children. I. A survey of professional workers. *Journal of Psychosomatic Research* 16**,** 7-18.

Conrad, C.D., LeDoux, J.E., Magarinos, A.M., and McEwen, B.S. (1999). Repeated restraint stress facilitates fear conditioning independently of causing hippocampal CA3 dendritic atrophy. *Behav Neurosci* 113(5)**,** 902-913.

Daviu, N., Fuentes, S., Nadal, R., and Armario, A. (2010). A single footshock causes long-lasting hypoactivity in unknown environments that is dependent on the development of contextual fear conditioning. *Neurobiol Learn Mem* 94**,** 183-190. doi: 10.1016/j.nlm.2010.05.005.

Hagenaars, M.A., Stins, J.F., and Roelofs, K. (2012). Aversive life events enhance human freezing responses. *Journal of Experimental Psychology: General* 141**,** 98-105. doi: 10.1037/a0024211.

Imanaka, A., Morinobu, S., Toki, S., and Yamawaki, S. (2006). Importance of early environment in the development of post-traumatic stress disorder-like behaviors. *Behav Brain Res* 173(1)**,** 129-137. doi: 10.1016/j.bbr.2006.06.012.

Liu, H., Atrooz, F., Salvi, A., and Salim, S. (2017). Behavioral and cognitive impact of early life stress: Insights from an animal model. *Prog Neuropsychopharmacol Biol Psychiatry* 78**,** 88-95. doi: 10.1016/j.pnpbp.2017.05.015.

Lukkes, J.L., Mokin, M.V., Scholl, J.L., and Forster, G.L. (2009). Adult rats exposed to early-life social isolation exhibit increased anxiety and conditioned fear behavior, and altered hormonal stress responses. *Hormones and Behavior* 55**,** 248-256. doi: 10.1016/j.yhbeh.2008.10.014.

Main, M., and Solomon, J. (1990). "Procedures for identifying infants as disorganized/disoriented during the Ainsworth Strange Situation," in *Attachment in the preschool years: Theory, research, and intervention,* eds. G.T. Greenberg, D. Cicchetti & E.M. Cummings. (Chicago: University of Chicago Press), 121-160.

Menard, J.L., Champagne, D.L., and Meaney, M.J.P. (2004). Variations of maternal care differentially influence 'fear' reactivity and regional patterns of cFos immunoreactivity in response to the shock-probe burying test. *Neuroscience* 129**,** 297-308. doi: 10.1016/j.neuroscience.2004.08.009.

Niermann, H.C.M., Figner, B., Tyborowska, A., van Peer, J., Cillessen, A.H.N., and Roelofs, K. (2017). Defensive freezing responses link Hypothalamic-Pituitary-Adrenal-axis activity and internalizing symptoms in humans. *Psychoneuroendocrinology* 82**,** 83-90. doi: 10.1016/j.psyneuen.2017.05.001.

Niermann, H.C.M., Ly, V., Smeekens, S., Figner, B., Riksen-Walraven, J.M., and Roelofs, K. (2015). Infant attachment predicts bodily freezing in adolescence: Evidence from a prospective longitudinal study. *Frontiers in Behavioral Neuroscience* 9**,** 263. doi: 10.3389/fnbeh.2015.00263.

Petersen, A.C., Crockett, L., Richards, M., and Boxer, A. (1988). A self-report measure of pubertal status: Reliability, validity, and initial norms. *J Youth Adolesc* 17(2)**,** 117-133. doi: 10.1007/bf01537962.

R Core Team (2017). "R: A language and environment for statistical computing", (ed.) R.F.f.S. Computing. (Vienna, Austria).

Rincón-Cortés, M., and Sullivan, R.M. (2016). Emergence of social behavior deficit, blunted corticolimbic activity and adult depression-like behavior in a rodent model of maternal maltreatment. *Translational psychiatry* 6(10)**,** e930.

Roelofs, K., Hagenaars, M.A., and Stins, J. (2010). Facing freeze: Social threat induces bodily freeze in humans. *Psychological Science* 21**,** 1575-1581. doi: 10.1177/0956797610384746.

Rubin, M. (2017). Do p values lose their meaning in exploratory analyses? It depends how you define the familywise error rate. *Review of General Psychology* 21**,** 269-275. doi: 10.1037/gpr0000123.

Sanders, B.J., and Knoepfler, J. (2008). Neonatal handling increases cardiovascular reactivity to contextual fear conditioning in borderline hypertensive rats (BHR). *Physiology & Behavior* 95**,** 72-76. doi: 10.1016/j.physbeh.2008.04.021.

Saranson, I.G., Johnson, J.H., and Siegel, J.M. (1978). Assessing the impact of life changes: Development of the life experiences survey. *Journal of Consulting and Clinical Psychology* 46**,** 932-946. doi: 10.1037/0022-006x.46.5.932.

Singmann, H., Bolker, B., and Westfall, J. (2015). afex: Analysis of factorial experiments. R package version 0.15-2.

Tyborowska, A., Volman, I., Smeekens, S., Toni, I., and Roelofs, K. (2016). Testosterone during Puberty Shifts Emotional Control from Pulvinar to Anterior Prefrontal Cortex. *J Neurosci* 36(23)**,** 6156-6164. doi: 10.1523/jneurosci.3874-15.2016.

van Bakel, H.J.A., and Riksen-Walraven, J.M. (2002). Quality of infant-parent attachment as reflected in infant interactive behaviour during instructional tasks. *Journal of Child Psychology and Psychiatry* 43**,** 387-394. doi: 10.1111/1469-7610.00029.

van den Berg, C.L., Lamberts, R.R., Wolterink, G., Wiegant, V.M., and van Ree, J.M. (1998). Emotional and footshock stimuli induce differential long-lasting behavioural effects in rats; involvement of opioids. *Brain Res* 799(1)**,** 6-15.

van Dijken, H.H., Mos, J., van der Heyden, J.A., and Tilders, F.J. (1992). Characterization of stress-induced long-term behavioural changes in rats: Evidence in favor of anxiety. *Physiol Behav* 52(5)**,** 945-951.

Varmuza, K., and Filzmoser, P. (2009). *Introduction to Multivariate Statistical Analysis in Chemometrics.* Boca Raton, Florida, US: CRC Press.

Yan, C.G., Rincon-Cortes, M., Raineki, C., Sarro, E., Colcombe, S., Guilfoyle, D.N., et al. (2017). Aberrant development of intrinsic brain activity in a rat model of caregiver maltreatment of offspring. *Transl Psychiatry* 7(1)**,** e1005. doi: 10.1038/tp.2016.276.

Yves, R. (2012). lavaan: An R package for structural equation modeling. *Journal of Statistical Software* 48(2)**,** 1-36.

1. All individuals (*n* = 100) having a least one freezing measure at ages 14 or 17 were included in these analyses. Of these 100 participants, 75% had freezing data at both assessment points (due to technical problems one of these participants had no heart rate data at age 14, and another one had no body sway data at age 17), while 21% had no freezing data at age 14 and 4% had no freezing data at age 17. Additionally, 6% of these participants had no data on early negative personal life events, and 5% on current negative personal life events. To verify whether the imputation strategy was appropriate to deal with missing values, we compared the results of the imputed models to the results of models where participants with missing values were removed. Similar significant and non-significant results were observed. [↑](#footnote-ref-1)
2. For exploratory and follow-up analyses, we did not control for multiple comparisons. [↑](#footnote-ref-2)
3. Data of all adolescents who participated in the assessments at either age 14 or age 17 were used to compute the correlations between heart rate and body sway difference scores separately per age. [↑](#footnote-ref-3)
